# Cavitation-driven bubble evolution and load mechanisms in particle-wall multiphase interactions^☆^

**DOI:** 10.1016/j.ultsonch.2025.107461

**Published:** 2025-07-08

**Authors:** Yuxuan Deng, Haiting Xi, Zhentao Gu, Xiaoming Yan

**Affiliations:** Bailie School of Petroleum Engineering, Lanzhou City University, Lanzhou 730071, PR China

**Keywords:** Bubble dynamic, Particle-fluid interaction, Cavitation, Structural response, Energy dissipation

## Abstract

The interaction among cavitation bubbles, particles, and solid boundaries critically governs energy transfer in fluid systems. This study employs high-resolution numerical simulations to investigate how fluid flow and solid surfaces influence small scale bubble dynamics, energy dissipation, and load effects. The simulations cover a range of initial particle-bubble distances (*D*_p_ = 0.5 to 1.2) and sinking velocities (*v*_p_ = 0.3 to 1.8), revealing how particle motion and boundary proximity influence bubble evolution and pressure loading. Results indicate that a single particle exposed to bubble collapse experiences intense water-jet impacts, producing unimodal pressure peaks exceeding 40 MPa. In contrast, near-wall scenarios generate bimodal pressure responses caused by bubble rupture and micro-jet formation, which concentrate collapse energy onto the particle; the secondary peak reaches approximately 20 MPa. At higher sinking velocities, particles penetrate the bubble, releasing energy as acoustic radiation and triggering complex oscillations. Although residual flow and pressure gradients continue to drive bubble shrinkage, jet formation is suppressed. Particle-bubble interactions modulate collapse behavior and attenuate water-jet loading on nearby solid boundaries. At lower velocities, delayed liquid film formation further reduces energy dissipation. These findings elucidate how boundary conditions and particle kinematics shape cavitation-induced energy transfer, offering insights into fluid–structure interactions relevant to acoustic erosion and sonochemical processes.

## 1 Introduction

Bubble dynamics has been a subject of intensive research across various engineering and scientific disciplines, particularly in the context of acoustic cavitation, where collapsing bubbles generate transient extreme conditions that drive sonochemical reactivity within fluid systems [1,2]. The behavior of bubbles plays a critical role in determining system efficiency and stability, especially through extreme conditions in collapsing bubbles, thus offering valuable insights for optimizing sonochemical processes, cavitation-assisted reactions, and energy transfer in industry [[3], [4], [5], [6]]. Under complex conditions, nonlinear cavitation bubble interactions become increasingly intricate, representing a core focus of bubble dynamics research. For instance, bubble collapse near solid surfaces generates high-impact micro-jets and shock waves, leading to material erosion [7]. In cavitation cleaning, the released energy intensifies chemical activity and enhances surface cleaning [8,9]. Investigating these processes enhances our understanding of bubble dynamics and provides insights into optimizing cavitation-driven chemical reactions and industrial applications.

Bubble-boundary interactions are fundamental to bubble dynamics, as rigid and elastoplastic surfaces distinctly influence both the formation of collapse jets and the propagation of shock waves. High-speed imaging and shadowgraphy have been used to capture these interactions [10]. Studies reveal that wall distance significantly affects bubble behavior and shock wave generation, enabling the development of predictive models for shock wave pressure and energy [11,12]. Research on bubble collapse on elastic surfaces has provided valuable insights into damage mechanisms and flow dynamics [13,14], while recent computational advances have facilitated high-resolution dynamic simulations of bubble evolution [[15], [16], [17]]. Based on the Rayleigh–Plesset equation and CFD simulations, bubble heat models estimate heat transfer and temperature in cavitation devices [18]. Cavitation models emphasize bubble–bubble interactions, particularly core size, while Kelvin impulse theory further elucidates fluid mechanics [19,20]. Furthermore, studies on cavitation bubble collapse have examined its impact on crystal structures, recrystallization, and rupture pressure dynamics, revealing that maximum pressure is localized at the base of the jet as it impinges on the wall [[21], [22], [23]]. In multiphase flows, the interaction between moving particles and bubbles affects medium stability and introduces nonlinear dynamics and flow-solid coupling. Experiments show that particle velocity is influenced by size, density, and proximity to the bubble, with the initial gap determining displacement [[24], [25], [26], [27]]. The modes of bubble collapse near particles are governed by dimensionless parameters, some of which redirect the collapse to shield adjacent walls [28,29]. Moreover, ultrasonic pulses can form and control stable aggregates of particles associated with larger carrier bubbles [30]. Numerical simulations are essential for quantifying bubble-particle interactions, revealing mechanisms critical for engineering applications. Potential flow, viscous, and compressible models help clarify how bubble collapse facilitates particle removal, thereby demonstrating key cavitation phenomena. Finite volume simulations of submerged spheres illustrate bacterial elimination via hydraulic cavitation and reveal micro-scale damage resulting from bubble–sphere interactions [31,32]. In flotation, pH variations reduce the zeta potential of coal particles and oily bubbles, while changes in gas flow underscore bubble expansion as the primary mechanism driving particle ejection in fluidized beds [33,34]. For low-rank coal particles, inverse induction time estimates for rising bubbles highlight the crucial role of the Reynolds number [35]. Nonetheless, controlling bubble behavior under complex boundary conditions remains a formidable challenge due to the intricate interplay of multiphase interactions, nonlinear dynamics, and variable environmental factors. To tackle these challenges, further refinement of experimental techniques and advanced numerical models is essential for accurately capturing bubble dynamics and ultimately optimizing cavitation-driven processes in practical applications. Part of the aforementioned studies have focused on the interaction between bubbles and individual elements, identifying key influencing factors and control parameters. Representative studies by [27,28,35], have investigated the interaction between fixed spherical boundaries and cavitation bubbles. Due to the high variability and boundary condition dependence of bubble-fluid coupling effects, there may be significant differences in bubble behaviour between fixed and moving boundaries. However, investigations into the interaction among cavitation bubbles, moving solids and fixed boundaries remain scarce, despite such scenarios being classical configurations where cavitation commonly occurs in fluid systems. This fluid–structure interaction process involves moving boundaries, elastic–plastic response, and non-linear bubble evolution. The mechanical mechanisms involved are more complex and diverse than those in the simple fluid–structure interaction scenarios described above.

**Fig. 1:**
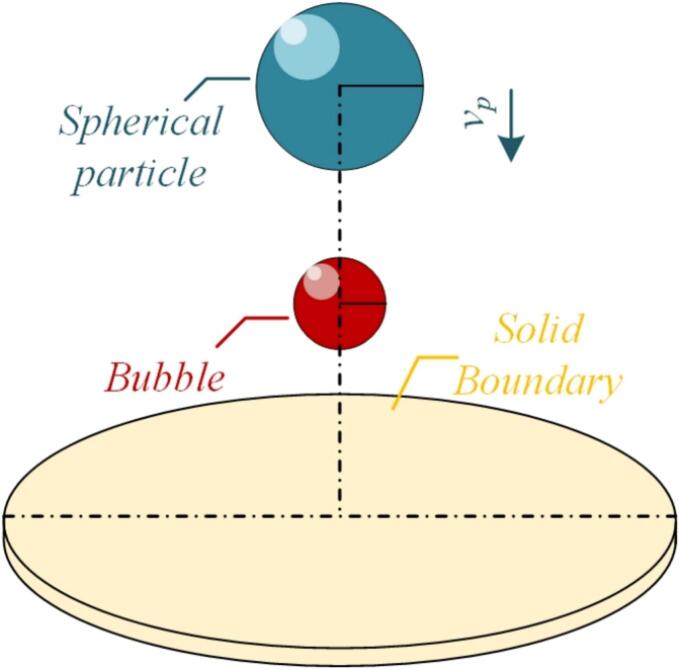
Schematic diagram of moving sphere-bubble-solid boundary interaction.

In this study, high-resolution numerical simulations are employed to explore the coupled dynamics among collapsing bubbles, sinking particles, and elastoplastic walls (Fig. 1). By systematically analyzing the influence of particle velocity and boundary proximity on bubble morphology, energy evolution, and load distribution, the results elucidate how confined geometries modulate cavitation dynamics. Particular attention is given to the mechanisms of bubble penetration, asymmetric collapse, jet formation, and energy transfer across multiphase interfaces. Unlike studies limited to free-field or rigid-wall conditions, this work highlights the complex feedback between mobile solid structures and evolving bubble interfaces under dynamic flow conditions. The findings deepen the understanding of bubble–structure interactions under realistic conditions and offer theoretical guidance for mitigating cavitation-induced damage, thereby laying the groundwork for future studies on ultrasound-driven bubble dynamics in chemically reactive or multiphase systems.

## 2 Methodology

In this study, numerical simulations were performed using ANSYS Autodyn, incorporating a multi-material Euler scheme and an Arbitrary Lagrangian-Eulerian (ALE) solver to model the direct coupling between boundaries, bubbles, and particles. These approaches enable accurate tracking of capture bubble interface evolution, shockwave propagation, and structural dynamics, offering a robust framework for understanding bubble-particle interactions in dynamic environments [36].

### 2.1 Calculation method

In our computational models, the Eulerian framework is adopted for the fluid domain to effectively resolve shockwave propagation and hydrostatic pressure effects. Compressibility is modeled through an appropriate equation of state, a multi-material Godunov scheme, extending the conventional Volume of Fluid (VOF) method, is utilized to improve interface tracking in multiphase systems [37]. To ensure precise delineation of phase boundaries and material transport, the Simple Line Interface Calculation (SLIC) method is applied [38]. Particle motion is simulated using Lagrangian elements, and ALE elements are employed for the aluminum alloy plate to better capture fluid–structure interactions under complex boundary conditions [39]. When deformation is negligible, the Lagrangian method uses a mesh that moves with the material. However, significant fluid motion may lead to severe mesh distortion or excessively stretched element aspect ratios. The Lagrangian-Eulerian solver serves as a key numerical approach in this study to model the direct coupling between boundaries, bubbles, and particles [40,41]. The ALE solver generates adaptive computational meshes that conform to the moving boundaries of material regions and employs boundary-fitted grids to discretize and solve the governing equations. This approach is particularly effective for simulating bubble–structure interaction problems. The governing partial differential equations, which express the conservation of mass, momentum, and energy, are formulated in the ALE coordinate system. Combined with appropriate material models and clearly defined initial and boundary conditions, these equations establish a comprehensive framework for the numerical solution of the problem. The ALE method combines the benefits of both Lagrangian and Eulerian approaches, allowing for flexible and accurate mesh reconfiguration during transient simulations. Free surfaces and material interfaces are handled with Lagrangian elements, while ALE re-meshing is limited to internal vertices, though planar surface nodes can also be processed. While the ALE approach minimizes the frequency of re-meshing required by pure Lagrangian methods, it cannot fully replace multi-material Eulerian solvers, especially in large-scale flow problems. The remap technique is employed to mitigate the effect of the detonation shockwave on sphere movement. A small equivalent charge is detonated in a 5 mm wedge-shaped fluid domain with a material outflow boundary. After the shockwave propagates, flow field data is recorded and mapped to the fluid domain containing the sphere and target plate. The turbulence model adopted in the numerical simulations is the SST k–ω model, a well-established and reliable approach known for its good accuracy in predicting boundary layer behavior and flow separation [42]. Detailed theoretical information is provided in Section 1.1 of the Supporting Information.

### 2.2 Mesh independence analysis

**Fig. 2:**
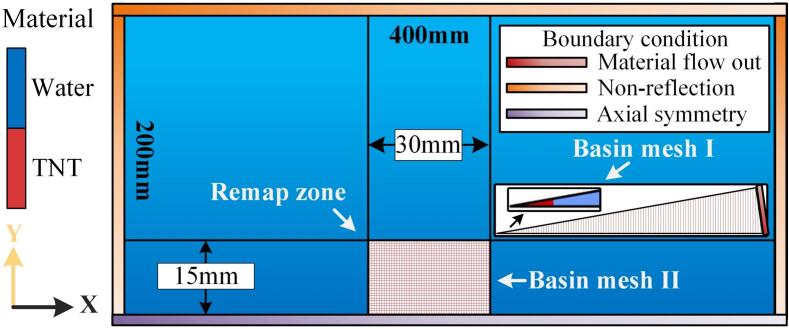
Spatial arrangement of the mesh configurations used in the simulation.

Simulating bubble dynamics and structural responses requires a numerical model with high spatial resolution and mesh precision. A mesh independence analysis was conducted to determine the optimal mesh size for the finite element (FE) model. Three distinct mesh configurations were evaluated: the wedge-shaped domain (Basin mesh I), the two-dimensional domain (Basin mesh II), and the aluminum alloy target plate mesh. AL7075-T6 is selected as the material for the target plate. This alloy possesses good machinability and high fatigue strength, making it widely used in high-stress structural applications. Its dynamic behavior is described by the Linear Equation of State (EOS) and the Johnson–Cook model. The Linear EOS effectively characterizes the pressure–density–energy relationship under high strain-rate impact, featuring a simple structure and numerical stability. The Johnson–Cook model accounts simultaneously for strain hardening and strain-rate strengthening, accurately predicting the plastic deformation behavior of the material under impact. The spatial arrangement of the domains is shown in Fig. 2, with the analysis centered on the refined mesh region in the two-dimensional domain.

**Fig. 3:**
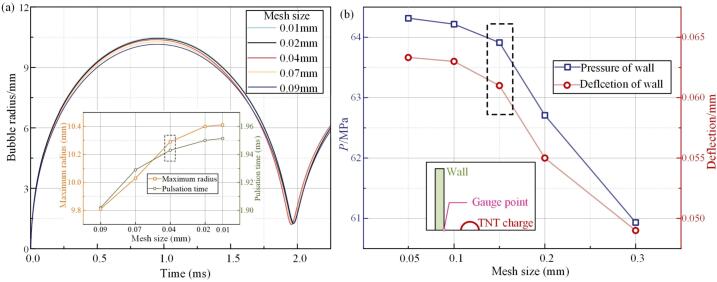
Verification of mesh independence (a) bubble pulsation and (b) wall pressure peak and deflection.

The pulsating behavior of a 0.003 g TNT explosion bubble in a free field was simulated using mesh sizes of 0.01 mm, 0.02 mm, 0.04 mm, 0.07 mm, and 0.09 mm. The relative errors between the calculated and theoretical values for the bubble's maximum radius and pulsation period are shown in Fig. 3 and Table S2 (Supporting Information). The theoretical values for the maximum radius and first pulsation period were 10.272 mm and 1.99 ms, respectively, validating the accuracy of the numerical method. To balance computational efficiency and accuracy, a 0.04  mm mesh (Basin mesh II) was selected, while a finer 0.02  mm mesh (Basin mesh I) was applied in the wedge-shaped model for more accurate flow field mapping. An explicit dynamics method was used in the simulation, with time steps regulated by the smallest mesh size to ensure stability. To assess the impact of mesh resolution on structural response, wall pressure and deformation of an aluminum alloy target plate under underwater explosion shockwaves were computed for various mesh sizes. Results exhibited convergence as the mesh refined from 0.3 mm to 0.05 mm, with stable outputs observed at 0.15 mm. The FE model therefore employed a 0.15 mm mesh for the target plate and a 0.2 mm mesh for the particle to optimize accuracy and computational efficiency.

The theoretical formula for UNDEX bubble pulsation is given by [43,44]:

(1) $$R_{m}=3.36\times {\left(\frac{W}{h+10.3}\right)}^{1/3}$$

(2) $$T=2.11\times \frac{W^{1/3}}{{\left(h+10.3\right)}^{5/6}}$$

The numerical uncertainty in this study was assessed using the Richardson Extrapolation Method. Specifically, the Grid Convergence Index (GCI) criterion was employed following the methodology presented in reference [45]. GCI estimates the discretization error of refined meshes based on results obtained from multiple grid levels. For the fluid domain, simulations were conducted using mesh sizes of 0.02 mm, 0.04 mm, and 0.07 mm. For the solid domain, mesh sizes of 0.1 mm, 0.15 mm, and 0.2 mm were used.

For the fluid domain, the convergence behavior of the maximum bubble radius and oscillation time is similar, with the bubble radius having a dominant influence on the simulation results. A numerical uncertainty analysis was conducted based on the maximum bubble radius obtained under different mesh resolutions, and the results are as follows:

(3) $$R_{\text{L}}=\frac{\phi_{\text{2}}-\phi_{\text{1}}}{\phi_{\text{3}}-\phi_{\text{2}}}\approx \text{0.507}$$

(4) $$p_{k}=\frac{\ln\left[\left(\phi_{3}-\phi_{2}\right)/\left(\phi_{2}-\phi_{1}\right)\right]}{\ln r_{\mathrm{eff}}}\approx 1.08$$

Assuming the theoretical order $p_{f}=2$. If $p_{f}>p_{k}$, the multiplier is 1.25.

(5) $$U_{\text{GCI}}=1.25\left|\delta_{p_{k}}\right|\approx 0.00089$$

For the solid domain, the convergence behavior of wall deflection and load peak values is similar, with the deflection directly reflecting the applied forces. A numerical uncertainty analysis was performed based on the wall deflection results obtained at different mesh resolutions, and the findings are as follows:

(6) $$R_{S}=\frac{\phi_{\text{2}}-\phi_{\text{1}}}{\phi_{\text{3}}-\phi_{\text{2}}}\approx \text{0.164}$$

(7) $$p_{k}=\frac{\ln\left[\left(\phi_{3}-\phi_{2}\right)/\left(\phi_{2}-\phi_{1}\right)\right]}{\ln r_{\mathrm{eff}}}\approx 5.26$$

If $p_{f}<p_{k}$, the multiplier is 1.25.

(8) $$U_{\text{GCI}}=1.25\left|\delta_{p_{f}}\right|\approx 0.642$$

According to the analysis, the numerical results for bubble oscillation exhibit low dispersion. The uncertainty in the solid domain deflection calculations is slightly higher but remains within an acceptable range, especially considering that the mechanical response of the wall is not the primary focus of this study.

The remap technique is used to transfer computational results from lower-dimensional fine-grid simulations to higher-dimensional domains. This method is widely applied in high-speed impact problems, as it improves computational efficiency and avoids excessive mesh refinement at early stages. In this study, the Euler Godunov solver incorporates the Block Remap interface to map flow field variables between dimensions, enabling accurate field reconstruction while mitigating mesh distortion. A more detailed description of the method can be found in [46].

### 2.3 Simulation model

**Fig. 4:**
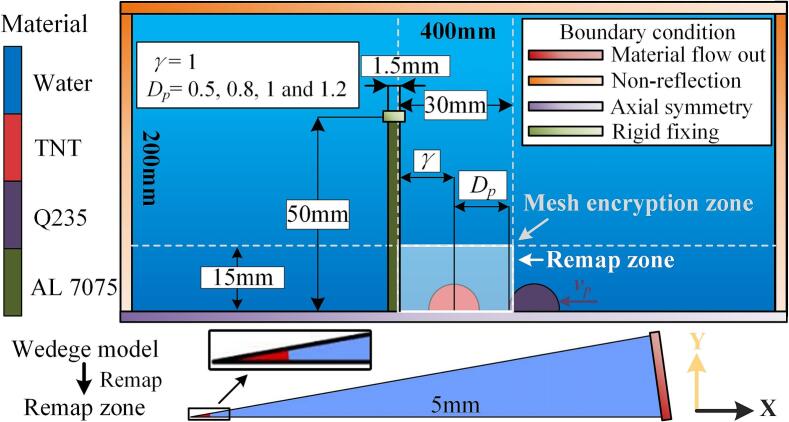
Computational domain diagram.

The numerical model consists of fluid and structure domains, modeled as a two-dimensional axisymmetric system (Fig. 4). The fluid domain dimensions are 0.2 m × 0.4 m, with a static pressure of 10.1325 kPa, simulating a 1-meter water depth. Non-reflective boundaries are applied on three sides, while the fourth is axisymmetric. The mesh refinement region is centered in the fluid domain, with a refined area of 0.03 m × 0.015 m, approximately 1.5 times the maximum bubble size. Gravitational acceleration is defined as 9.81 m/s^2^, directed along the negative X-axis. In the structural domain, the aluminum alloy target plate has a radius of 50 mm and a thickness of 1.5 mm, with rigid constraints applied at its edges. The particle, with a radius of 5 mm (half the maximum bubble radius), moves along the X-axis with an initial velocity (*v*_p_), impacting the target plate. The distance from the sphere's surface to the explosion center is denoted as *D*_p_. Based on these quantities, the relationships between the dimensionless distance *γ*, dimensionless distance *D*_p_, and dimensionless velocity *v*_p_ are established, as shown in the following formula:

(3) $$D_{p}=\gamma =\frac{d}{R_{m}}$$

(4) $$v_{p}=\frac{v}{R_{m}/T}$$

Where *d* is distance from the ball's surface to the center of bursting, and *v* is velocity of particle. The reference value of *v*_p_ is the average expansion velocity of the bubble in the first cycle in a free field.

### 2.4 Verification of numerical method

**Fig. 5:**
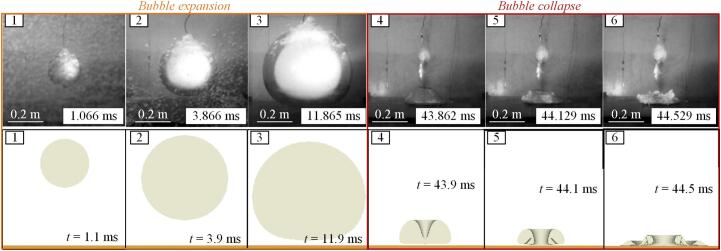
Comparison of simulation and experimental results [47]. The simulation closely matches the experimental observations of bubble expansion, migration, collapse, and water jet formation near the solid boundary.

To validate the numerical method for modeling bubble-boundary interactions, simulation results were compared with experimental data [47], as shown in Fig. 5. The experiment was conducted in a 2 m × 2 m × 2 m steel water tank filled with tap water, featuring three acrylic observation windows. The central window (0.4 m × 0.7 m) enabled visualization of bubble dynamics, while side windows were used to provide uniform illumination for imaging. Bubble generation was achieved using a PETN composite explosive (70 % PETN, 20 % aluminum powder, and 10 % inhibitor), with each charge weighing 4.0 g, encapsulated in a cylindrical wax shell (15 mm diameter, 15 mm height), corresponding to a TNT equivalent mass of 5.2  g. The explosion was initiated by an electric detonator. A high-speed camera behind the central window recorded bubble dynamics at 7500 frames per second, with 800 × 800-pixel resolution and a 1.0 m × 1.0 m field of view. The minimum resolvable unit was 1.25 mm per pixel, with an error of 0.34 % to 0.42 % for the maximum bubble radius. Time measurements had an interval of 0.133 ms, with a maximum error of 0.32 % relative to the initial bubble (approximately 42 ms). Comparison of simulation and experimental results shows that the numerical method accurately reproduces bubble expansion, deformation, collapse, and water jet formation near the solid boundary. The agreement, shown in Table S2, further confirms the robustness and reliability of the numerical method.

### 2.5 Computational assumptions of numerical method

To clarify the computational scope, several assumptions were adopted in this study: First, the simulations are conducted under an axisymmetric assumption, which simplifies the computational domain and reduces the cost, but also limits the ability to capture inherently three-dimensional behaviors such as asymmetric jetting and non-uniform bubble deformation. Second, fluid viscosity is neglected in the governing equations. While this may underestimate near-wall shear dissipation, the early-stage dynamics of bubble collapse are dominated by inertial and pressure-driven effects, making the inviscid approximation acceptable for capturing the primary flow features. Third, fluid compressibility is incorporated through an equation of state, which enables shock wave resolution and pressure field evolution. However, detailed modeling of acoustic wave attenuation and high-frequency energy dissipation remains beyond the current scope.

## 3 Results and discussion

Bubble motion near boundaries is influenced by boundary distance, local material properties, and geometry, resulting in nonlinear variations in expansion and collapse that affect fluid continuity and interaction. This study investigates how the sphere's initial velocity and its distance from both the bubble and boundary affect bubble evolution, loading, and energy transfer. At high Reynolds numbers, bubble motion is primarily governed by inertial forces, with the fluid approximated as inviscid and irrotational.

### 3.1 Single-particle and near-wall particle comparison

**Fig. 6:**
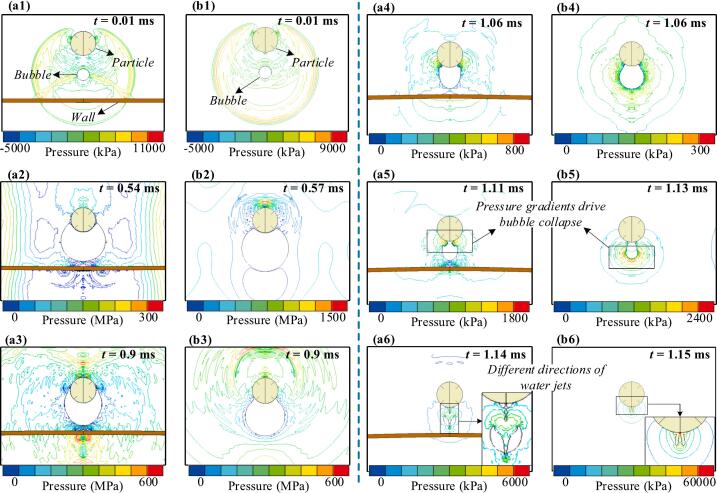
Comparison of flow field pressure contour plots for bubble motion in the single-particle and particle–wall scenarios. (a) Near-wall particle scenario and (b) Single-particle scenario.

We compare the fluid–structure interactions of a bubble near a single particle and between a particle and a wall, with particular attention to the distinct Bjerknes effects described as the net force acting on an oscillating bubble in a pressure gradient, which can drive migration toward or away from boundaries. Fig. 6 shows the evolution of bubble morphology and flow field pressure in both scenarios, with *v*_p_ = 1, *D*_p_ = 1, and λ = 1. Initially, the bubble's center of mass acts as the origin of shock waves. Upon contact with the solid boundary, wave transmission, reflection, and diffraction are induced. The reflected wave propagates back into the fluid domain and inhibits the bubble’s expansion in the direction of the wall. In contrast, when the bubble is positioned between the particle and the wall, reflected waves from both sides strongly constrain its expansion along the longitudinal direction (from the particle's center to the wall's center), as shown in Fig. 6a1 and b1.

**Fig. 7:**
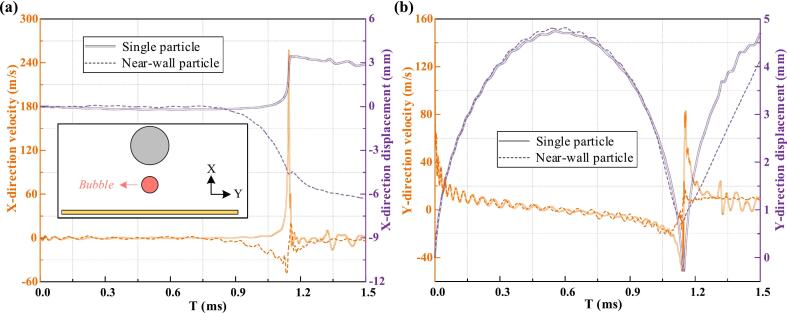
Comparison of the bubble's motion history in single-particle and particle–wall scenarios. (a) Bubble velocity and displacement along the X-axis, and (b) Bubble velocity and displacement along the Y-axis.

After reaching maximum volume, the bubble undergoes over-expansion followed by rapid contraction, driven by the pressure gradient. The presence of the wall restricts expansion, embedding the particle more deeply and amplifying the vortex effect along its surface (Fig. 6a2 and b2). The wall also accelerates bubble growth and influences the liquid film dynamics, altering bubble indentation and increasing energy dissipation. During contraction, the anisotropic pressure gradient drives non-spherical deformation and jet formation (Fig. 6a3 to 6a5, 6b3 to 6b5). Collapse starts at high-curvature regions: in the single-particle case, it occurs away from the particle, while the wall suppresses rapid contraction. According to potential flow theory, during collapse the vortex component of the bubble located beneath the particle moves rapidly along the particle’s surface. This rapid motion can cause local pressure spikes, which in turn create a positive feedback loop that enhances the bubble's collapse in that vicinity. This causes the water jet to aim toward the particle in the single-particle case and toward the wall, with a secondary jet toward the particle in the near-wall case. The motion history of the bubble in both scenarios is shown in Fig. 7. Although bubble motion remains largely comparable in both scenarios they diverge substantially once collapse begins. In the single-particle case, the bubble translates toward the particle due to buoyancy-induced pressure gradients and boundary effects. Although gravity acts in the opposite direction, the dominant driving force arises from the asymmetry in the surrounding flow field. In the near-wall case, stronger wall attraction leads to marked downward motion and a water jet directed toward the wall. Consequently, the increased energy dissipation during collapse weakens the water jet’s acceleration in the near-wall scenario, resulting in a lower peak velocity compared to the single-particle case.

**Fig. 8:**
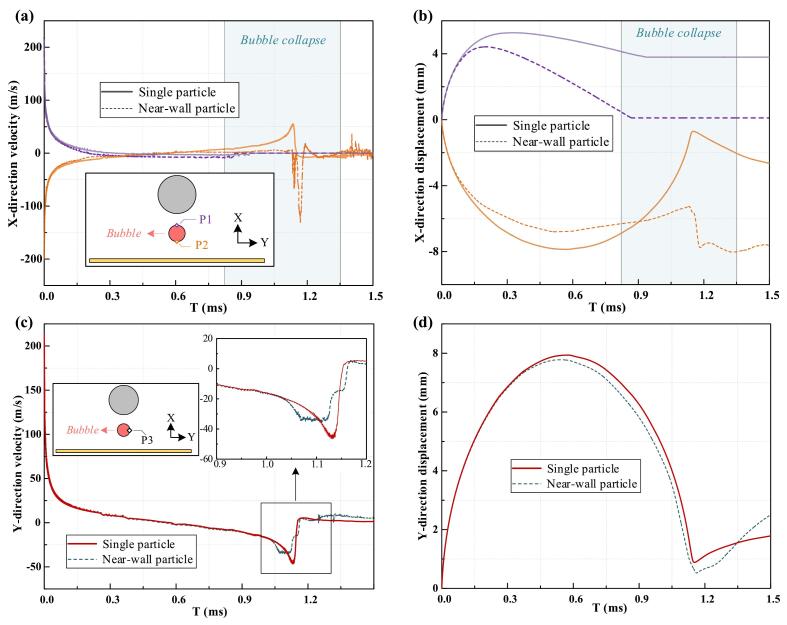
Comparison of the bubble's motion history under single-particle and particle–wall scenarios. (a) Velocity and (b) displacement at monitoring points P_1_ and P_2_, (c) velocity and (d) displacement at monitoring point P_3_.

To investigate bubble evolution, monitoring points (P_1_, P_2_, and P_3_) were placed at the bubble's longitudinal boundaries (Fig. 8). At P_1_ and P_2_, the upper portion of the bubble reaches maximum displacement earlier than full expansion due to localized compression by the particle. In contrast, the lower surface reaches maximum displacement only at full volume. The near-wall condition further inhibits bubble growth, resulting in even earlier maximum displacement at both points. At P_1_, rapid contraction and flow reversal near the vortex at the particle's lower surface. Meanwhile, the velocity peak at P_2_ is even more notable under the near-wall condition, with water jet timing differing significantly between the collapse phase (single-particle case) and the rebound phase (near-wall case). Additionally, at P_3_, the particle and wall boundaries constrain lateral expansion, producing a smaller lateral dimension than in the single-particle scenario. Expansion and contraction are primarily driven by inertial forces and modulated by the flow field's pressure gradient. During collapse, the velocity peak at P_3_ is higher in the single-particle case but shorter in duration, whereas under near-wall conditions, the peak is lower but sustained longer due to positive feedback from localized high-pressure regions. Finally, the minimum displacement at P_3_ is smaller in the near-wall scenario as bubble tearing, the fragmentation of the bubble interface due to asymmetric collapse or external flow gradients, occurs.

**Fig. 9:**
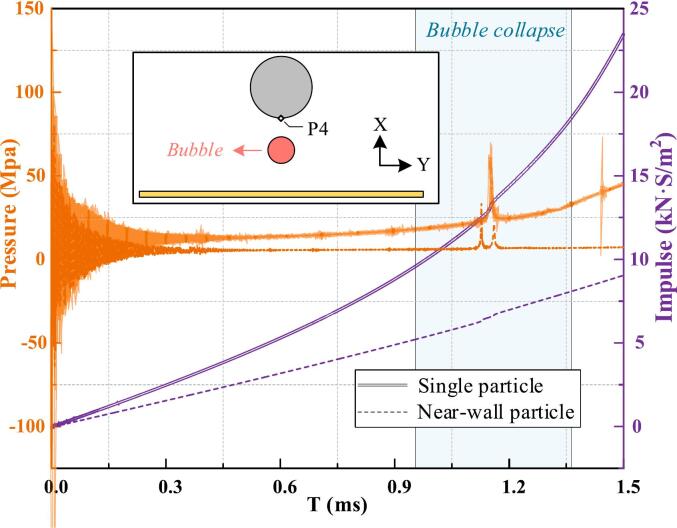
Comparison of the load history at the bottom monitoring point P_4_ in both scenarios.

Kinetic and total energy histories of the bubble under two boundary conditions are compared (Fig. S1, Supporting Information). After bubble formation, the expansion rate decreases sharply, and energy exchange between the bubble and the flow field rapidly declines. Unlike shock waves, the residual flow persists with relatively low energy levels. Before collapse, kinetic and total energy remain similar in both scenarios. However, upon rupture, bubble tearing and water jet formation induce high-energy responses, with reversible energy stored in surrounding liquid particles redirected back into the bubble as the residual flow reverses. In the single-particle case, the bubble absorbs significantly more energy during collapse than in the near-wall case, where bubble tearing releases intense shock waves that dissipate energy as acoustic radiation, thereby reducing the bubble's kinetic energy. Bubble-particle interactions are bidirectional, with the Bjerknes effect, wake flow, and shock loading jointly influencing the particle's sinking speed. Fig. 9 shows the load history at the bottom center (P_4_) under single-particle and near-wall conditions. Impulse, representing the integral of pressure over time, quantifies the load experienced by the particle. The particle experiences a higher impulse load throughout the cycle in the single-particle case due to more pronounced wake effects. During collapse, the single-particle scenario yields a single high-energy shock, whereas the near-wall condition produces a double-peaked pressure history due to bubble tearing and micro-jet formation.

### 3.2 Characteristics of bubble evolution

**Fig. 10:**
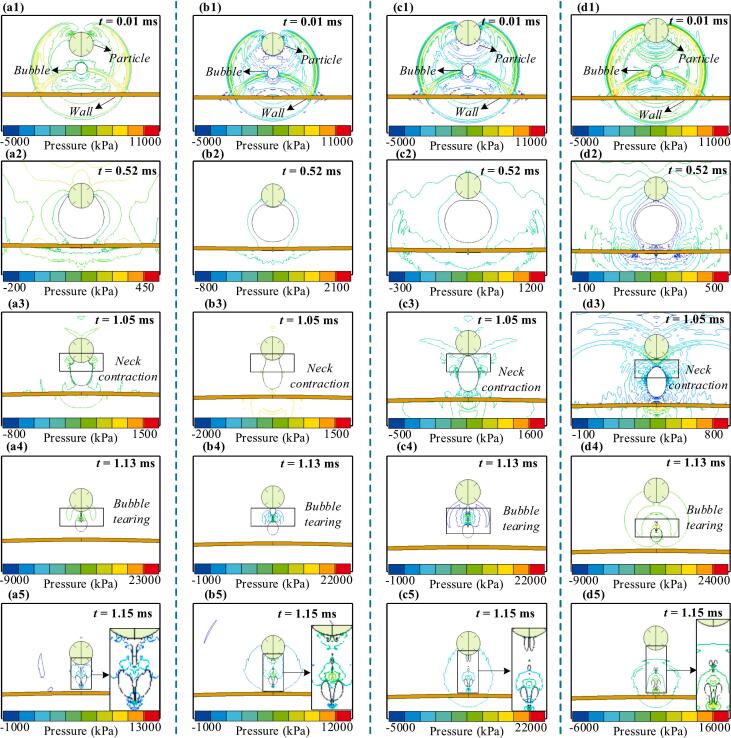
Particle sinking with low initial velocity and bubble evolution under different initial distances, with flow field pressure contours. (a) *D*_p_ = 0.5, (b) *D*_p_ = 0.8, (c) *D*_p_ = 1, (d) *D*_p_ = 1.2. A small initial distance leads to vortex formation on the particle surface, shifting the neck contraction point upward and causing more significant rupture. A larger initial distance results in downward movement of both the neck contraction point and rupture location.

The sinking particle and fixed wall boundary conditions result in complex and coupled bubble evolution dynamics. This section examines bubble behavior under both varying initial distances and particle sinking velocities. Fig. 10 presents bubble evolution and flow field pressure contours for different initial distances with a low particle sinking velocity (*v*_p_ = 0.3). When the initial distance is small (Fig. 10a and b), a vortex forms along the particle's lower surface during bubble expansion. As the bubble expands and the particle sinks, the vortex gradually migrates upward along the particle surface. During collapse, the boundary velocity reverses, creating a reverse vortex. According to flow theory, convergence at the bubble neck drives rupture, and stronger vortex intensity caused by deeper particle immersion results in rupture occurring closer to the particle surface. In contrast, when no significant vortex forms or the bubble does not contact the particle (Fig. 10c and d), the pressure gradient accelerates lateral contraction, while the Bjerknes effects from the particle and wall jointly inhibit axial contraction. Because the particle's Bjerknes influence is weaker than that of the wall, the bubble tends to rupture in closer proximity to the particle.

Fig. S2 (Supporting Information) shows bubble evolution and flow field pressure contours at four initial distances for a moderate particle sinking velocity (*v*_p_ = 1). At *D*_p_ = 0.5, the expanding bubble flows over the particle's surface and reaches its upper side. The relatively fast sinking particle induces localized tearing at the bubble front during contraction, producing a high-pressure zone (Fig. S2a3) that facilitates reverse vortex flow. As the initial distance increases to *D*_p_ = 1.2, the bubble contacts the particle's lower surface during contraction. The Bjerknes effect inhibits axial collapse, leading to the formation of a hammer-shaped structure at the bubble's upper part. Due to limited vortex activity, the rupture occurs farther from the particle than in Fig. S2b and 2c, with a larger portion of the separated bubble still remaining near the particle (Fig. S2d4). Fig. S3 (Supporting Information) shows the evolution of the bubble and flow field pressure contours under various initial distances when the particle sinks at a higher velocity (*v*_p_ = 1.8). At *D*_p_ = 0.5, a strong vortex forms along the particle's lower surface during expansion and converges toward the tail. However, the fast-speed sinking particle pierces the bubble before it collapses, transforming it into a toroidal shape and forming a double-connected flow domain (Fig. S3a3). This rupture releases substantial energy as acoustic radiation, resulting in complex post-collapse oscillations. As the bubble contracts, it fails to generate a high-speed water jet. The particle effectively dissipates the collapse energy, reducing impact on nearby solid boundaries. At *D*_p_ = 0.8, the particle no longer pierces the bubble but partially detaches it, forming a ring-shaped structure that flows around the particle's tail. The remaining bubble migrates toward the wall and undergoes collapse and rebound. Although no jet forms, the separated bubble can still impose loading on the wall during rebound. As the initial distance increases to *D*_p_ = 1, the tearing effect weakens, allowing the residual bubble to retain more energy and produce stronger rebound-induced pressure pulses toward the wall (Fig. S3c5). When *D*_p_ = 1.2, the ring-shaped rupture disappears entirely. The bubble migrates toward the wall with reverse flow along the particle's lower surface, causing rupture and producing both pressure waves and generating both pressure waves and water jets (Fig. S3d5).

**Fig. 11:**
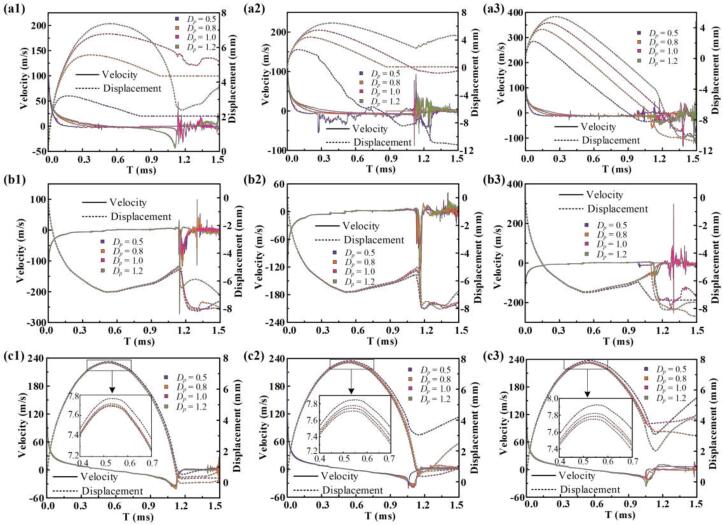
Comparison of the bubble motion histories under varying initial distances and particle sinking velocities of 0.3, 1, and 1.8, respectively. (a) Velocity and displacement at the bubble's upper surface pole (P_1_), (b) Velocity and displacement at the bubble's lower surface pole (P_2_), and (c) Velocity and displacement at the bubble's side surface pole (P_3_).

Fig. 11 tracks the motion histories of the bubble's upper surface pole (P_1_), lower surface pole (P_2_), and side surface pole (P_3_) under varying initial distances and particle sinking velocities. In all cases, increasing the initial distance provides more space for expansion, leading to greater maximum displacement and a longer time to reach the peak position. At P_1_, the velocity peak occurs when the water jet pierces the separated bubble after rupture, with the highest value observed at a particle velocity of *v*_p_ = 1. In contrast, at *v*_p_ = 1.8, jetting is less pronounced, and no distinct velocity spike is recorded. At P_2_, the wall-induced Bjerknes effect remains consistent across all cases, resulting in similar maximum vertical positions (Fig. 11b). However, significant differences emerge after rupture. At *v*_p_ = 0.3 and 1, strong rebound jets form toward the wall, producing sharp velocity spikes. At *v*_p_ = 1.8 and a short initial distance of *D*_p_ = 0.5, no jet is clearly observed, and no distinct peak appears. Nevertheless, a rebound-induced pressure pulse still produces a moderate velocity rise at P_2_. At P_3_, lateral bubble growth is less sensitive to particle motion than axial growth but still exhibits variation (Fig. 11c). As the initial distance decreases, more energy is redirected laterally, increasing the bubble'.

s maximum horizontal spread. When *D*_p_ = 0.5, the presence of both the particle and the wall imposes significant constraints on the axial growth of the bubble. As a result, a larger portion of the bubble’s energy is redirected toward lateral expansion. Under this condition, a distinct flow separation region forms beneath the particle, leading to more pronounced rebound dynamics after the bubble collapses.

### 3.3 Load and energy transfer characteristics

Complex boundary conditions affect bubble morphology, energy transfer within the flow field, and the resulting loading on both the particle and the wall. This section examines the influence of varying particle sinking velocities and initial distances on these dynamic behaviors.

**Fig. 12:**
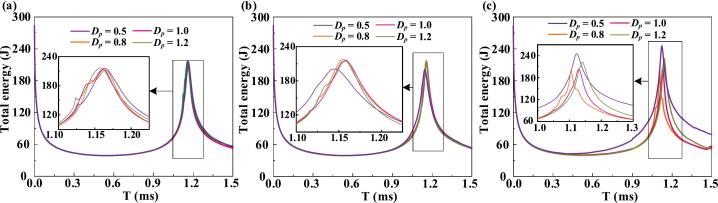
Total energy history of the bubble at various initial distances and particle sinking velocities. (a) *v*_p_ = 0.3, (b) *v*_p_ = 1.0, (c) *v*_p_ = 1.8.

Fig. 12 presents the total energy evolution of the bubble from its formation to the first collapse under three sinking velocities and four initial distances. During formation and oscillation, energy exchange primarily occurs through the bubble boundary, driving fluid motion in the surrounding domain. At low sinking velocities, the particle's influence on the bubble interface is minimal, resulting in a limited effect on energy transfer. However, as the sinking velocity increases, notable differences in energy history emerge due to enhanced interaction with the bubble boundary. These local interactions alter expansion and collapse behavior, sometimes causing ring-shaped ruptures or even bubble penetration, thereby significantly modulating the energy exchange between the bubble and the surrounding fluid.

**Fig. 13:**
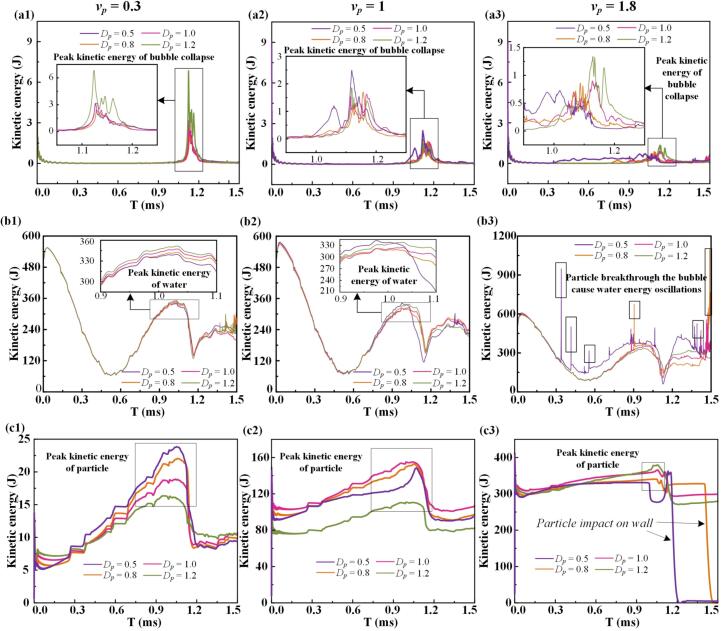
Kinetic energy histories at different initial distances and particle sinking velocities of 0.3, 1, and 1.8, respectively. (a) Bubble: Due to the relatively slow approach velocity of the low-speed sinking particle, the liquid film forms later at larger initial distances, leading to weaker dissipation of kinetic energy. (b) Fluid: When the liquid film forms earlier and persists longer, enhanced dissipation of kinetic energy is observed. (c) Particle: During bubble collapse, the particle is influenced by both gravity and the residual flow field, and the majority of its kinetic energy gain originates from the reversible energy stored in the surrounding fluid.

The bubble's energy is primarily internal, while its kinetic energy offers a more direct indicator of motion. Fig. 13a presents the bubble's kinetic energy history under different initial distances and particle sinking velocities of 0.3, 1, and 1.8. As noted by Abdal [48], the liquid film between the bubble and particle drains when they approach, leading to dissipation of kinetic energy. At a sinking velocity of 0.3, larger initial distances result in higher peak kinetic energy during the collapse and rebound, as the slower particle approach delays liquid film formation and reduces energy dissipation. At *v*_p_ = 1, intense vortex formation leads to partial rupture and promotes boundary collapse (Fig. S2a3). When the sinking velocity increases to 1.8, the particle pierces the bubble, leading to greater energy loss and a reduced kinetic energy peak. Fig. 13b shows the kinetic energy evolution of the surrounding fluid. During the early stage of bubble formation, shock waves are emitted, and momentum is transferred from the bubble to the liquid. As expansion slows, energy exchange diminishes until collapses. During collapse, the compressing water gains kinetic energy. At *v*_p_ = 0.3, shorter initial distances result in earlier film formation and lower energy peaks. At *v*_p_ = 1, rupture-induced shock waves raise the water's kinetic energy. At *v*_p_ = 1.8, bubble penetration by the fast-sinking particle strongly accelerates the surrounding fluid, increasing its kinetic energy. Fig. 13c presents the particle's kinetic energy evolution. During expansion, pressure from the interface resists particle motion, while during collapse, reversed fluid flow drives downward acceleration. Following impact with the wall, the particle's kinetic energy initially decreases, followed by a rebound-induced increase. The particle's energy history is closely coupled to that of the surrounding fluid. Compared to Fig. 13b, the particle's peak kinetic energy during collapse follows an opposite trend, modulated by initial distance. Strong vortex strength can accelerate the particle, while penetration or ring rupture may lead to reduced acceleration.

**Fig. 14:**
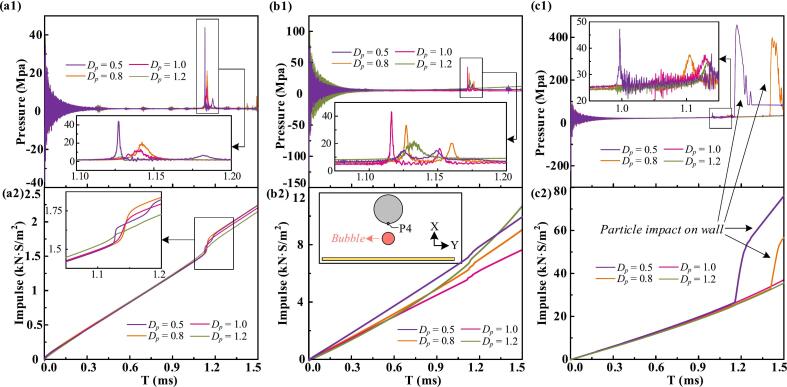
The load history at the gauge point P4 on the bottom surface of the particle for different initial distances, with particle sinking initial velocities. (a) *v*_p_ = 0.3, (b) *v*_p_ = 1.0, (c) *v*_p_ = 1.8.

As shown in Fig. 14, the bubble exerts load on the particle through three primary mechanisms: the formation of a water jet directed at the particle, shock waves released during bubble rupture, and compression from the rebounding bubble. The specific loading behavior varies under different conditions and significantly shapes the pressure history at P_4_. At particle sinking velocities of 0.3 and 1, distinct pressure peaks are observed during collapse. When strong shock waves from bubble rupture act on the particle's lower surface, a double-peak pressure signature is observed, consisting of a higher amplitude component associated with bubble collapse and a lower impulse due to its brief duration. These conditions share a common feature: when the bubble ruptures, a large portion of the separated bubble remains adsorbed on the particle's bottom surface, and the size of this residual bubble directly modulates the magnitude of the load. At a sinking velocity of 1.8, the particle pierces the bubble, allowing the water jet to form without restriction. However, the complex wave interactions generated at the rupture site during penetration lead to substantially higher pressure loads at P_4_.

## 4 Conclusion

This study investigates the fluid–structure interaction between sinking particles, bubbles, and thin walls using numerical simulations. The effects of particle and wall boundary conditions on bubble evolution, load, and energy transfer are analyzed. The main findings are as follows:

(1) Under single-particle conditions, the particle experiences a high-energy shock from the water jet during bubble collapse, resulting in a single-peak pressure profile. In near-wall scenarios, the pressure history shows a double-peak response due to the high-energy shock and micro-jet from the split bubble directed at the particle.
(2) At sufficiently high sinking velocities, the particle penetrates the bubble, releasing significant energy that dissipates as acoustic radiation and induces complex wave interactions. After the bubble is pierced, it continues to contract under residual flow and the pressure gradient but loses the ability to generate high-speed water jets. The particle thus diminishes the bubble's collapse potential, reducing the impact of the collapse jet on the solid boundary.
(3) For particles sinking at low velocities, the slower approach delays liquid film formation, which in turn leads to lower energy dissipation when the initial distance is large.
(4) Variations in particle kinetic energy are strongly correlated with the kinetic energy of the surrounding fluid. During bubble collapse, the particle is accelerated by both the gravitational field and residual flow, with the increase in kinetic energy primarily sourced from the reversible energy stored within the surrounding liquid.
(5) Future work will focus on extending the current axisymmetric model to full three-dimensional (3D) simulations, which can capture azimuthal instabilities and asymmetrical jetting phenomena that are not resolved under the axisymmetric assumption. In addition, the effects of fluid viscosity will be incorporated to improve the accuracy of modeling energy dissipation, vortex shedding, and the damping behavior observed during bubble collapse near compliant structures. Further, exploring alternative particle materials with varied density, elasticity, and surface wettability could provide insights into material-dependent bubble-particle–wall interactions.
(6) These advancements are expected to contribute to a deeper understanding of multi-physics coupling in cavitating environments and offer potential applications in designing cavitation-resistant materials, precision particle manipulation in fluid systems, and enhancing sonochemical reactor performance by leveraging controlled jet collapse and localized pressure amplification.

Prime Originality Statement.

The submitted content is original and does not involve plagiarism or copyright infringement. The authors have completed the work seriously, and it has not been previously published or under consideration for publication elsewhere. All authors approve the submission, which, if accepted, will not be published in the same form, in English or any other language, without the written consent of the publisher.

## CRediT authorship contribution statement

**Yuxuan Deng:** Writing – original draft, Software, Methodology. **Haiting Xi:** Writing – review & editing, Investigation. **Zhentao Gu:** Supervision, Investigation. **Xiaoming Yan:** Supervision.

## Declaration of competing interest

The authors declare that they have no known competing financial interests or personal relationships that could have appeared to influence the work reported in this paper.
