# Supplementary material for: Cavitation-driven bubble evolution and load mechanisms in particle-wall multiphase interactions
Source: Ultrason Sonochem. 2025 Jul 8;120:107461. doi: 10.1016/j.ultsonch.2025.107461 (PMC12284372; doi:10.1016/j.ultsonch.2025.107461)
Supplement: Supplementary Data 1 [file mmc1.docx]

**Supplementary material**

**Cavitation-driven bubble evolution and load mechanisms in particle-wall multiphase interactions**

Yuxuan Deng^a,*^, Haiting Xi^a^, Zhentao Gu^a^, Xiaoming Yan^a^

*^a^* *Bailie School of Petroleum Engineering, Lanzhou City University, Lanzhou 730071, P R China.*

1. **Computational methodology**

In the numerical calculation process, the polynomial EOS is used to describe the water medium. The EOS expression is:

| $\left\{\begin{matrix} p=A_{1}\mu+A_{2}\mu^{2}+A_{3}\mu^{3}+(B_{0}+B_{1}\mu) \text{ρ}_{0}e , \mu<0 \\ p=T_{1}\mu+T_{2}\mu^{2}+B_{0}\text{ρ}_{0}e , \mu>0 \end{matrix}\right.$ | (1) |
| --- | --- |
| $p=T_{1}\mu+T_{2}\mu^{2}+B_{0}\rho_{0}e$ | (2) |
| $\mu=\frac{\rho}{\rho_{0}}-1$ | (3) |
| $e=\frac{(\rho gH+p_{atm}\text{）}}{\rho}B_{0}$ | (4) |

Where$A_{1}$, $A_{2}$, $A_{3}$, $B_{0}$, $B_{1}$, $T_{1}$ and $T_{2}$ are constants; $\rho$ is density of water; $\rho_{0}$ is initial density of water; $H$ is depth of water; $p_{atm}$ is atmospheric pressure; and $e$ is specific internal energy. The parameters of the aqueous medium at a water depth of 1 m are shown in Table 1.

The ideal gas EOS is used to describe the air medium:

| $p=\text{（}\gamma-1\text{）}\rho e$ | (5) |
| --- | --- |

The gas density is represented by $\rho=1.225\times10^{\text{-3}}\text{g/cm}^{3}$; the adiabatic index is $\gamma\text{=1.4}$; and the specific internal energy is $e=2.068\times10^{5}\text{kJ/kg}$. Under this parameter, the air pressure is standard atmospheric pressure (101.325kPa).

The JWL EOS is used to describe the pressure of detonation products:

| $p=A(1-\frac{\omega}{R_{1}V})e^{-R_{1}V}+B(1-\frac{\omega}{R_{2}V})e^{-R_{2}V}+\frac{\omega E}{V}$ | (6) |
| --- | --- |

Where $E$ is the specific internal of TNT; *V* is relative specific volume of the detonation products; and $A$, $B$, $R_{1}$, $R_{2}$, $\omega$are constants. When the explosive expands to a specific volume, its behavior can be accurately described using the ideal gas EOS. The relevant material properties can be found in the Autodyn material library.

Q235 is selected as the material of particle. The structure is rigid and does not plastically deform or fail under external excitation, and $\rho=\text{7.83g/cm}^{3}$。

AL7075-T6 is selected as the material of target plate. Its dynamic behavior is described by the Linear EOS and Johnson-Cook model:

| $\sigma=\text{（}A+B\varepsilon_{P}^{n}\text{）}[1+Cln(\varepsilon^{\cdot} /\varepsilon^{\cdot} _{0})](1-T_{H}^{m})$ | (7) |
| --- | --- |
| $T_{H}=(T-T_{room})/(T_{melt}-T_{room})$ | (8) |

Where$A$ represents the static yield limit; $B$ and $n$ are strain hardening parameters; $C$ is the strain rate-dependent parameter; $\varepsilon_{P}^{n}$​ is the effective plastic strain; $\varepsilon^{\cdot}$​ is the effective plastic strain rate; $\varepsilon^{\cdot} _{0}$​ is the quasi-static strain rate; $T_{melt}$​ is the melting point of the metal; $T_{room}$ is the room temperature; and $m$ is the temperature softening parameter. The relevant material properties can be found in the Autodyn material library. The parameters of AL7075-T6 are listed in Table S1.

**Table S1:** Parameters of water, TNT and AL 7075-T6

| Water | | TNT | | AL 7075-T6 | |
| --- | --- | --- | --- | --- | --- |
| Parameter | value | Parameter | value | Parameter | Value |
| $A_{1}\text{/GPa}$ | 2.20 | $A\text{/GPa}$ | 373.77 | *A*/MPa | 495 |
| $A_{2}\text{/GPa}$ | 9.54 | $B\text{/GPa}$ | 3.75 | *B*/MPa | 303.6 |
| $A_{3}\text{/GPa}$ | 1.46 | $R_{1}$ | 4.15 | *C* | 0.0097 |
| $B_{0}$ | 0.28 | $R_{2}$ | 0.9 | *n* | 0.39 |
| $B_{1}$ | 0.28 | $\omega$ | 0.35 | *m* | 0.77 |
| $T_{1}\text{/GPa}$ | 2.20 | $E\text{/(GJ/m}^{3}\text{)}$ | 7.0 |  | 635 |
| $T_{2}\text{/Pa}$ | 0 | $\rho\text{/}(g\text{/}cm^{3})$ | 1.63 |  | 294 |
| $\rho_{0}\text{/(g/cm}^{3}\text{)}$ | 1.0 | $p_{\text{cJ}}/\text{GPa}$ | 21.0 | $\rho\text{/}(g\text{/}cm^{3})$ | 2.804 |
| $e\text{/(J/kg)}$ | 361.875 | $D_{\text{cJ}}\text{/(km/s)}$ | 6.93 | *K*/GPa | 69.9 |

**Table S2:** Comparison of the bubble parameters between simulation and analysis.

| Mesh size | 0.02mm | 0.04mm | 0.07mm | 0.09mm |
| --- | --- | --- | --- | --- |
| Relative error of *R_m_* | 1.25% | 0.18% | 2.36% | 4.51% |
| Relative error of *T* | 2.01% | 2.36% | 3.06% | 4.42% |

**Fig. S1.** Comparison of the total energy and kinetic energy history of the bubble in both scenarios. (a) Kinetic energy history of the bubble and (b) Total energy history of the bubble. During bubble collapse, the jet formed under the single-particle boundary condition significantly increases the bubble's kinetic and total energy. In contrast, although a water jet and tearing also occur in the near-wall particle scenario, the increase in kinetic energy is minimal, and the change in total energy is substantially lower than in the single-particle case.

**Fig. S2.** The evolution of the bubble and the flow field pressure contour maps for different initial distances when the particle sinks with a moderate initial velocity. (a) *D*_p_ = 0.5, (b) *D*_p_ = 0.8, (c) *D*_p_ = 1, (d) *D*_p_ = 1.2. At a sinking speed of 1, the bubble experiences more compression from the particle's lower surface during expansion. For smaller initial distances, the bubble can move over and approach the upper half of the particle’s surface. As the initial distance increases, the bubble’s vortex behavior diminishes, and the longitudinal length during neck contraction becomes longer.

**Fig. S3.** The evolution of the bubble and the flow field pressure contour maps for different initial distances when the particle sinks with a highly initial velocity. (a) *D*_p_ = 0.5, (b) *D*_p_ = 0.8, (c) *D*_p_ = 1, (d) *D*_p_ = 1.2. At a sinking velocity of 1.8, the bubble is pierced by the particle, triggering complex wave dynamics under small initial distance conditions. High-speed water jets are only formed when the initial distance is sufficiently large.
